# Altered uterine contractility in response to β-adrenoceptor agonists in ovarian cancer

**DOI:** 10.1007/s12576-016-0500-1

**Published:** 2016-11-12

**Authors:** Beata Modzelewska, Maciej Jóźwik, Marcin Jóźwik, Stanisław Sulkowski, Anna Pędzińska-Betiuk, Tomasz Kleszczewski, Anna Kostrzewska

**Affiliations:** 1 Department of Biophysics, Medical University of Białystok, Mickiewicza 2A, 15-089 Białystok, Poland; 2 Department of Gynecology and Gynecologic Oncology, Medical University of Białystok, Białystok, Poland; 3 Department of Gynecology, Gynecologic Endocrinology and Obstetrics, Faculty of Medical Sciences, University of Warmia and Mazury, Olsztyn, Poland; 4 Department of General Pathomorphology, Medical University of Białystok, Białystok, Poland; 5 Department of Experimental Physiology and Pathophysiology, Medical University of Białystok, Białystok, Poland; 6 Łomza Medical College of the Universal Educational Society, Łomża, Poland

**Keywords:** β_2_-Adrenoceptors, β_3_-Adrenoceptors, Cervical cancer, Endometrial cancer, Ovarian cancer, Uterine contraction

## Abstract

We aimed to prospectively examine β-adrenoceptor-mediated uterine contractility in women suffering from gynecological malignancies. Myometrial specimens were obtained from non-pregnant women undergoing hysterectomy for benign gynecological disorders, and ovarian, endometrial, synchronous ovarian–endometrial, and cervical cancer. Contractions of myometrial strips in an organ bath before and after cumulative dosages of β_2_- and β_3_-adrenoceptor agonists with preincubation of propranolol, SR 59230A, and butoxamine were studied. All agonists induced a dose-dependent attenuation for uterine contractility in endometrial or cervical cancer, similar to that observed in the reference group. Contradictory effects were observed for ovarian cancer alone or in combination with endometrial cancer. CL 316243 or ritodrine abolished the relaxation, whereas BRL 37344 increased the uterine contractility in ovarian cancer. Moreover, β-adrenoceptor antagonists caused varied effects for β_2_- or β_3_-adrenoceptor agonists. Our experiments demonstrate that ovarian cancer, alone or as synchronous ovarian–endometrial cancer, substantially alters uterine contractility in response to β-adrenoceptor agonists.

**Electronic supplementary material:**

The online version of this article (doi:10.1007/s12576-016-0500-1) contains supplementary material, which is available to authorized users.

## Introduction

There is a growing body of evidence that adrenergic signaling, a central mediator of stress, affects numerous cellular processes that are also critical for carcinogenesis [1–3]. Specifically, adrenergic signaling can affect an extensive range of cancer-related molecular pathways via regulation of β-adrenoceptor-bearing tumor cells and other cells present in the tumor’s microenvironment [2, 4].

Many reports have demonstrated that agonists of both β_2_- and β_3_-adrenoceptors cause concentration-dependent relaxation of the contractile activity of human and animal myometrium [5–11]. β_2_-Adrenoreceptor agonists, such as ritodrine or salbutamol, are the most commonly used tocolytic agents. However, their benefits are limited because of associated significant adverse cardiovascular effects [12, 13]. In turn, there is in vitro evidence that the potential cardiovascular effects of β_3_-adrenoreceptor agonists is less than that of β_2_-adrenoreceptor agonists [14]. Nevertheless, before β_3_-adrenoreceptor agonists become therapeutic drugs with a potential target for tocolysis, it is vital to obtain more comprehensive studies on functions mediated by this receptor subtype in humans [15].

Although the innervation of the ovaries by vagal parasympathetic and sympathetic nerves has been described, the involvement of these autonomic nerves in ovarian function has not yet been clarified [16]. Moreover, β-adrenoceptors have been identified on several types of cancer cells, including ovarian cancer cell lines [17], yet little is known about their effects on uterine contractility in gynecological cancers. In fact, our careful literature review indicates that this topic has not yet been explored, despite the significant progress observed in the past decade in research linking bio-behavioral factors with tumor progression.

Studies accomplished to date aimed to indicate that spontaneous and rhythmic contractions of the non-pregnant uterus have an important role in human reproduction, predominantly for fertilization and embryonic implantation [18]. Postmenopausal uterine contractility is somewhat less pronounced [19]. However, disruption of physiological characteristics in uterine contractility is observed in a wide variety of clinical disorders, including dysmenorrhea, endometriosis, spontaneous and recurrent abortion [20], and most likely in cancers. It has been suggested that attention paid to symptoms related to abnormal uterine contractility may help in early application of non-invasive diagnostic procedures [21].

Our previous observations, together with literature data concerning the effect of stress on carcinogenesis, have led us to examine, in a systematic prospective way, the β-adrenoceptor-mediated response of uterine contractility in women affected by an array of gynecological malignancies.

## Materials and methods

### Human myometrial tissue

Myometrial specimens were obtained from non-pregnant women undergoing hysterectomy for: (1) benign gynecological disorders (*n* = 41; aged 39–71 years, median 49; reference group), (2) epithelial ovarian cancer (*n* = 20; aged 35–74 years, median 51), (3) synchronous primary cancer of the endometrium and ovary (*n* = 3; aged 41–64 years, median 58), (4) endometrial cancer (*n* = 25; aged 49–83 years, median 62), and (5) cervical cancer (*n* = 6; aged 40–56 years, median 49). Premenopausal patients were operated in their follicular phase of menstrual cycles. The participating women were carefully informed about the nature of the study and gave informed written consent in advance with the local Institutional Review Board having earlier approved the study.

Surgical indications for benign gynecological disorders included: dysfunctional bleeding (*n* = 8), uterine leiomyoma(s) (*n* = 19), cervical pre-malignancy (intraepithelial neoplasia) (*n* = 10), ovarian serous cystadenoma (*n* = 2), and ovarian fibroma (*n* = 2). All specimens underwent a detailed pathological review by the fourth author (S.S.), and a final diagnosis was given according to the World Health Organization criteria for pathology and genetics of tumors of female genital organs [22]. Histologic types of ovarian cancer were as follows: serous (*n* = 12), mucinous (*n* = 2), endometrioid (*n* = 2), and mixed serous and endometrioid (*n* = 4). The histologic composition of synchronous cancers was: endometrioid in both the uterus and ovary (*n* = 2), and mixed endometrioid and squamous cell components in the uterus and the endometrioid type in the ovary (*n* = 1). All endometrial cancers were of endometrioid type; all cervical cancers were of squamous cell type.

### Sample processing

A myometrial tissue block was transversely excised from the uterine fundus with preliminary macroscopic inspection and subsequent histopathological examination of the circumferential margin of resected specimens for the presence of tumor tissue or other abnormalities. The myometrial samples were placed in ice-cold Tyrode’s solution and immediately transferred to the laboratory where they were processed as previously described [23]. Briefly, on average 10–12 strips of 10 mm × 1 mm × 1 mm were obtained under a dissecting microscope. The strips were then mounted in a thermostatically controlled isolated organ bath containing 20 ml of Tyrode’s solution at 37 °C, pH 7.4, and bubbled with carbogen (95% O_2_ + 5% CO_2_). Tyrode’s solution consisted of (mmol/l): NaCl 139.6; KCl 2.68; MgCl_2_ 1.05; NaH_2_PO_4_ 1.33; CaCl_2_ 1.80; NaHCO_3_ 25.0; and glucose 5.55. The strips were left for an equilibration period of 1–2 h, within which the passive tension was adjusted to 2 mN. The bath solution was changed every 15 min. After equilibration, regular phasic contractions were achieved.

### Data acquisition

Myometrium activity was recorded by a force transducer with digital output (BIO-SYS-TECH, Białystok, Poland) and with the DASYLab software unit (version 9.0; Laboratory Data Acquisition System, SuperLogics, Waltham, MA, USA). Before each experiment, strips were activated by 80 mmol/l K^+^. Only strips showing a stable response to potassium were used in the experiments. BRL37344, CL316243 (both: β_3_-adrenoceptor selective agonists), or ritodrine (β_2_-adrenoceptor selective agonist) was added cumulatively to the organ chambers (range 10^−10^–10^−4^ mol/l) at 10-min intervals, and the effects were recorded. SR 59230A (β_3_-adrenoceptor selective antagonist), butoxamine (β_2_-adrenoceptor selective antagonist), and propranolol (β_1_- and β_2_-adrenoceptor antagonist), each at a concentration of 10^−6^ mol/l, were added to the organ bath 20 min before the administration of the agonists. The responses were quantified by the area under the curve (AUC), amplitude, frequency of active contractions, and basal tension. The AUC reflected the total quantity of changes over time representing the contractile activity of myometrial strips before and after the administration of the given drug. The AUC was measured as the area under all recorded contractions over a 10-min interval before the addition of a β-adrenoceptor agonist [24].

### Drugs and solutions

Drugs and reagents were purchased from a number of sources: BRL 37344 sodium (sodium;2-[4-[2-[[2-(3-chlorophenyl)-2-hydroxyethyl]amino]propyl]phenoxy] acetate), ritodrine hydrochloride, propranolol hydrochloride, and butoxamine hydrochloride were obtained from Sigma-Aldrich (St. Louis, MO, USA); CL 316243 (disodium 5-[(2*R*)-2-[[(2*R*)-2-(3-chlorophenyl)-2-hydroxyethyl]amino]propyl]-1,3-benzodioxole-2,2-dicarboxylate hydrate) and SR 59230A hydrochloride (2*S*)-1-(2-ethylphenoxy)-3-[[(1*S*)-1,2,3,4-tetrahydronaphthalen-1-yl]amino]propan-2-ol hydrochloride) were from Tocris Bioscience (Bristol, UK).

Stock solutions of β-adrenoceptor agonists or antagonists were prepared daily using bidistilled water. Series of dilutions were prepared on the day of experiment and were maintained at room temperature throughout the duration of the experiment. All substances were added directly to the organ bath containing Tyrode’s solution, which was also made on a daily basis.

### Measurement of contraction parameters

Responses to agonists were calculated as percent (%) inhibition of spontaneous contractions of the myometrial strips before β-adrenoceptor agonist administration. Mean concentration response curves to agonists were analyzed by fitting to a four-parameter logistic equation using non-linear regression (PRISM 6.0, GraphPad Software Inc., San Diego, CA, USA). The AUC was evaluated by calculating the integral of the appropriate section of the curve. The maximal response (*E*_max_) was expressed as a percentage of the contractile activity before administration of β-adrenoceptor agonists, whereas the concentrations of agents that resulted in a half-maximal effect were expressed as log EC_50_ [23].

### Statistical analysis

All results were expressed as mean ± SEM with “*n*” denoting the number of experiments performed on myometrial strips from different patients. Dose–response was determined using analysis of variance (ANOVA) followed by a non-parametric or parametric Dunnett’s multiple comparison test where appropriate. All analyses were performed using Prism 6 for Windows (version 6.0, GraphPad Software Inc., San Diego, CA, USA). Values were considered to be statistically significant at *p* < 0.05.

## Results

All experiments were performed on myometrial strips exhibiting regular spontaneous contractile activity. Spontaneous contractility was present in 27 (79.4%) patients from the reference group (aged 39–69 years; median 48.0); 12 (63.2%) from the ovarian cancer group (aged 35–71 years; median 47.5), all three from the synchronous ovarian–endometrial cancer group (aged 41–64 years; median 58.0), 16 (64.0%) from the endometrial cancer group (aged 49–76 years; median 60.5), and five (83.3%) from the cervical cancer group (aged 40–56 years; median 44.0). As an internal quality measure, all tissues were examined histopathologically at the circumferential margin for the presence of malignancy. One specimen from the endometrial cancer group was found to be infiltrated with malignant cells and thus excluded from further studies. Therefore, the endometrial cancer group consisted of 15 patients (aged 50–76 years; median 62.5), contributing 56.0% of the examined specimens.

All examined strips presented a similar basal tension (*p* > 0.05): in the reference group 1.98 ± 0.56 mN (*n* = 27); ovarian cancer 1.99 ± 0.81 mN (*n* = 12); synchronous ovarian–endometrial cancer 1.98 ± 0.80 mN (*n* = 3); endometrial cancer 1.96 ± 0.98 mN (*n* = 15), and cervical cancer 1.97 ± 0.71 mN (*n* = 5). The mean amplitude of the spontaneous uterine contractility in the synchronous cancer group (2.52 ± 0.31 mN) was significantly smaller than in both reference (3.91 ± 0.21 mN; *p* = 0.0038) and endometrial cancer (3.62 ± 0.21 mN; *p* = 0.031) groups. There were no statistical differences in the amplitude for the other groups (ovarian cancer 3.90 ± 0.32 mN; cervical cancer 3.41 ± 0.21 mN). The mean frequency of spontaneous contractions per 10 min in the reference group (3.80 ± 0.21) was significantly smaller than in the synchronous ovarian–endometrial cancer and cervical cancer groups (4.32 ± 0.31; *p* = 0.011 and 4.11 ± 0.21; *p* < 0.0001, respectively). Moreover, the frequency observed in ovarian cancer (3.90 ± 0.21) was smaller than in cervical (4.11 ± 0.21; *p* < 0.0001) but higher than in endometrial cancer (3.21 ± 0.20; *p* < 0.001). Successively, the mean frequency in synchronous ovarian–endometrial cancer was significantly higher as compared to that in endometrial cancer (*p* = 0.011). In turn, the contraction duration in the reference group (87.10 ± 3.31 s; *n* = 33) was significantly shorter (*p* < 0.0001) than in ovarian cancer (129.90 ± 4.61 s), synchronous ovarian–endometrial cancer (139.21 ± 11.00 s), or endometrial (149.01 ± 5.82 s) or cervical (100.00 ± 4.61 s, *n* = 5) cancer. Additionally, the contraction duration observed in cervical cancer was markedly shorter as compared to ovarian (*p* < 0.0001), synchronous ovarian–endometrial (*p* = 0.002), or endometrial cancer (*p* < 0.0001).

### Effects of β-adrenoceptor agonists on spontaneous myometrial contractions

**Fig. 1:**
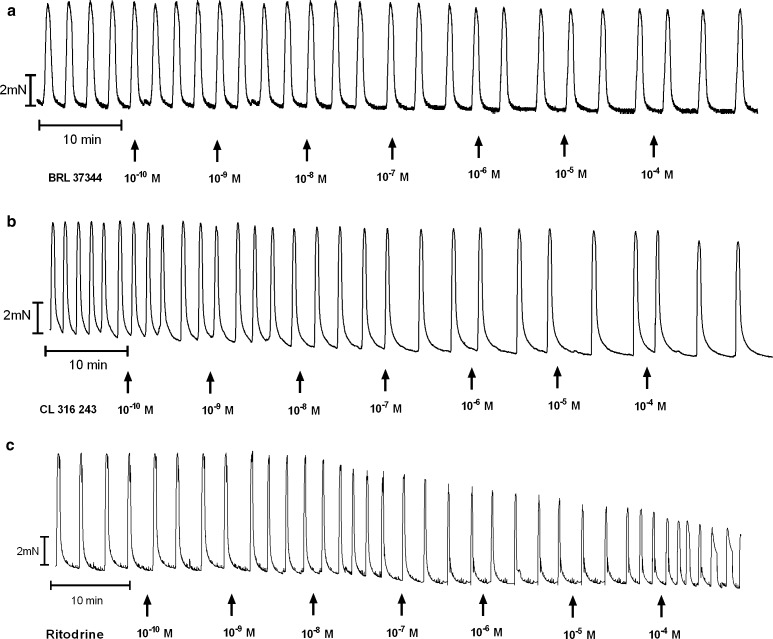
A representative tracing of the effects of cumulatively administered BRL 37344 (**a**), Cl 316,243 (**b**), and ritodrine (**c**) on spontaneous contractions of human non-pregnant myometrial strips from the reference group

**Fig. 2:**
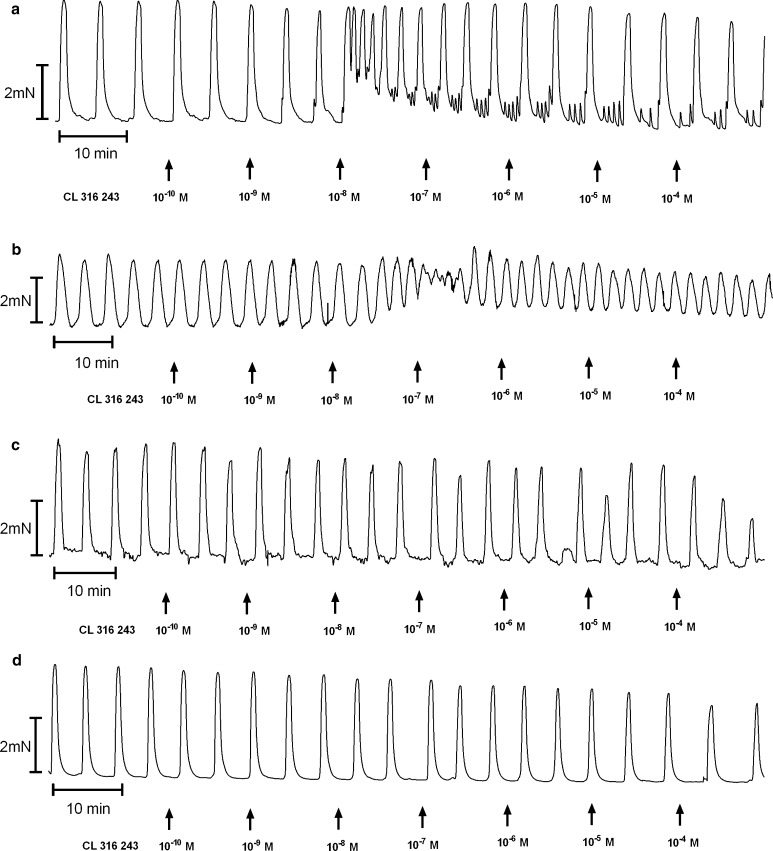
An original tracing of spontaneous contractile activity of human non-pregnant myometrial strips and the effects of cumulatively administered Cl 316 243 in the ovarian (**a**), synchronous ovarian–endometrial (**b**), endometrial (**c**), and cervical cancer (**d**) groups

The cumulative addition of BRL 37344, CL 316243 or ritodrine in concentrations higher than 10^−10^ mol/l resulted in a concentration-dependent decrease for the uterine contractility observed as statistically significant reduction for the AUC in the reference, endometrial and cervical cancer groups (Figs. 1, 2c, d, 4a, 5a, 6a; Table 1).

**Table 1:** Log EC_50_ and *E*_max_ for BRL 37344, CL 316243, or ritodrine on spontaneous uterine contractility of the human non-pregnant myometrium

Groups	β-antagonist	β-agonist								
		BRL 37344			CL 316243			Ritodrine		
		LogEC_50_	*E*_max_ (%)	*n*	logEC_50_	*E*_max_ (%)	*n*	logEC—_50_	*E*_max_ (%)	*n*
Reference	None	−7.0 ± 0.2	69.1 ± 1.7	12	−7.2 ± 0.1	66.9 ± 1.3	15	−7.6 ± 0.1	57.6 ± 1.5	12
	Propranolol	−7.1 ± 0.1	66.8 ± 1.2	10	−7.6 ± 0.1	63.3 ± 1.1	10	−8.5 ± 0.5	93.6 ± 0.9	10
	SR 59230A	−5.7 ± 0.4	86.8 ± 1.9	11	−5.6 ± 0.3	91.0 ± 1.5	11	−6.6 ± 0.1	60.5 ± 1.2	10
	Butoxamine	−7.0 ± 0.1	69.5 ± 1.2	10	−7.2 ± 0.1	66.4 ± 2.2	10	−6.5 ± 0.4	85.7 ± 1.6	10
Ovarian cancer	None	−7.4 ± 0.7	118.3 ± 4.5	10	–	98.1 ± 5.9	10	−7.6 ± 1.8	102.8 ± 2.9	10
	Propranolol	–	97.2 ± 1.3	9	−6.3 ± 3.6	100.2 ± 2.8	9	−5.5 ± 0.8	113.7 ± 6.9	9
	SR 59230A	−7.9 ± 1.3	95.5 ± 2.1	10	–	95.8 ± 1.8	9	−5.9 ± 0.6	90.3 ± 3.0	9
	Butoxamine	−8.1 ± 0.6	92.4 ± 4.5	8	–	102.2 ± 4.8	8	−5.5 ± 1.2	106.4 ± 2.5	9
Synchronous ovarian and endometrial cancer	None	−5.5 ± 1.6	99.0 ± 2.6	3	−8.3 ± 3.7	107.0 ± 4.8	3	−9.2 ± 0.6	97.5 ± 1.1	3
	Propranolol	−6.6 ± 0.9	94.4 ± 1.3	3	−6.0 ± 1.3	108.9 ± 6.3	3	−5.9 ± 1.4	98.8 ± 1.3	3
	SR 59230A	−7.2 ± 0.6	93.6 ± 1.2	3	–	93.6 ± 1.3	3	−6.1 ± 1.1	94.0 ± 6.3	3
	Butoxamine	−7.7 ± 1.8	98.4 ± 1.6	3	−7.7 ± 0.9	96.2 ± 0.3	3	−7.0 ± 2.8	100.0 ± 3.1	3
Endometrial cancer	None	−7.5 ± 0.2	67.8 ± 2.2†	7	−7.2 ± 0.3	68.5 ± 2.4	7	−7.5 ± 0.4	66.6 ± 3.1*	9
	Propranolol	−7.2 ± 0.2	71.4 ± 2.0†	7	−7.2 ± 0.2	61.7 ± 2.2	7	−8.3 ± 0.4	92.3 ± 0.8	10
	SR 59230A	−5.6 ± 0.5	87.0 ± 3.1	7	−5.2 ± 0.5	90.2 ± 2.4	7	−7.4 ± 0.2	65.9 ± 1.5	7
	Butoxamine	−7.5 ± 0.2	67.3 ± 1.8	8	−7.0 ± 0.2	68.4 ± 2.1	8	−6.7 ± 0.3	86.7 ± 1.0	10
Cervical cancer	None	−7.0 ± 0.2	66.5 ± 2.4	5	−7.5 ± 0.2	69.0 ± 1.7	5	−8.2 ± 0.2	64.9 ± 2.5	5
	Propranolol	−7.7 ± 0.1	60.8 ± 1.4	5	−7.2 ± 1.3	65.6 ± 1.6	5	−8.3 ± 0.6	90.2 ± 1.6	5
	SR 59230A	−5.5 ± 0.4	83.0 ± 2.7	5	−6.05 ± 0.7	90.3 ± 2.	5	−6.9 ± 0.2	64.2 ± 1.8	5
	Butoxamine	−7.1 ± 0.2	68.4 ± 1.7	5	−7.0 ± 0.3	66.9 ± 3.1	5	−5.9 ± 0.2	84.8 ± 1.1	5

LogEC_50_ is the logarithm of concentrations of agents that resulted in a half-maximal effect

*E*_max_ is the maximal response, expressed as a percentage of the contractile activity before administration of β-adrenoceptor agonists

The values are mean ± SEM of *n* individual myometrial strips from different patients

The effects caused by the cumulative addition of β-adrenoceptor agonists on ovarian and synchronous ovarian–endometrial cancer were quite contradictory to those observed in the reference group. CL 316243 or ritodrine, in concentrations higher than 10^−8^ and 10^−9^ mol/l, respectively, did not relax (*p* < 0.001) the myometrial strips as compared to the reference group (Figs. 2a, b, 5a, 6a; Table 1). BRL 37344 in concentrations higher than 10^−8^ mol/l caused a significant increase of AUC value in ovarian cancer (*p* < 0.001) (Fig. 4a; Table 1). In synchronous ovarian–endometrial cancer, there was a lack of muscular relaxation which was statistically significant for concentrations of this β_3_-adrenoceptor agonist higher than 10^−8^ mol/l, when compared to the reference group (*p* < 0.001) (Fig. 4a; Table 1).

### Effects of β-adrenoceptor antagonists on β-adrenoceptor agonists-induced relaxation of spontaneous myometrial contractile activity

#### Propranolol

The addition of propranolol to the organ bath, as previously shown [7], did not significantly alter the concentration–response curve for BRL 37344 in the reference and endometrial cancer groups. However, a statistically significant shift of the curve to the left was observed for cervical cancer (*p* < 0.05) (Fig. 4b; Table 1).

**Fig. 3:**
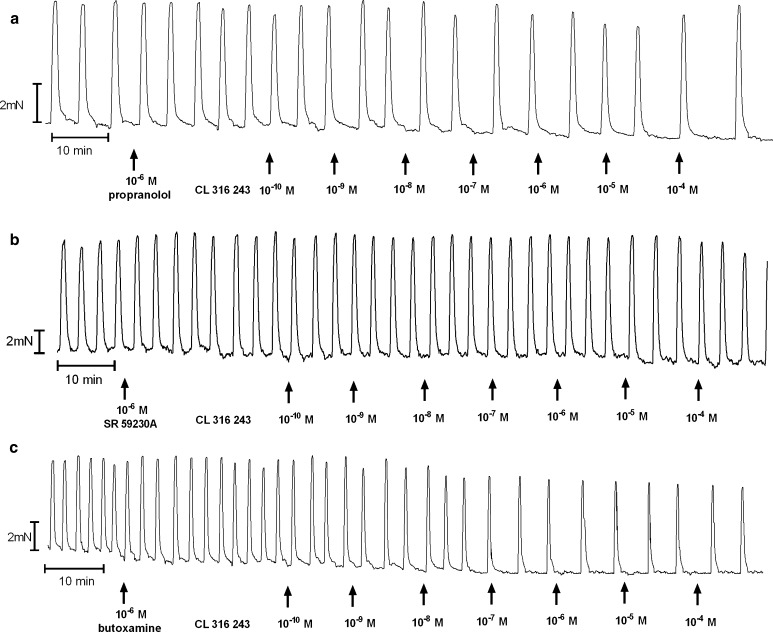
An original tracing of the effects of cumulatively administered Cl 316,243 after preincubation with propranolol (**a**), SR 59230A (**b**), or butoxamine (**c**) on spontaneous contractions of human non-pregnant myometrial strips from the reference group

Preincubation with propranolol did not alter the concentration–response curve for CL 316243 in the reference, endometrial cancer, and cervical cancer groups (Figs. 3a, 5b). There was no significant variation in the mean value of log EC_50_ and *E*_max_ for CL 316243 in the absence or presence of propranolol in the endometrial or cervical cancer groups, however, a significant (*p* < 0.05) leftward shift of the concentration–response curve for the reference group was observed (Table 1).

Preincubation of myometrial strips with propranolol caused a significant inhibition of the concentration–response curve for ritodrine in concentrations higher than 10^−9^ mol/l (*p* < 0.001) in the reference, endometrial cancer, and cervical cancer groups (Fig. 6b; Table 1).

**Fig. 4:**
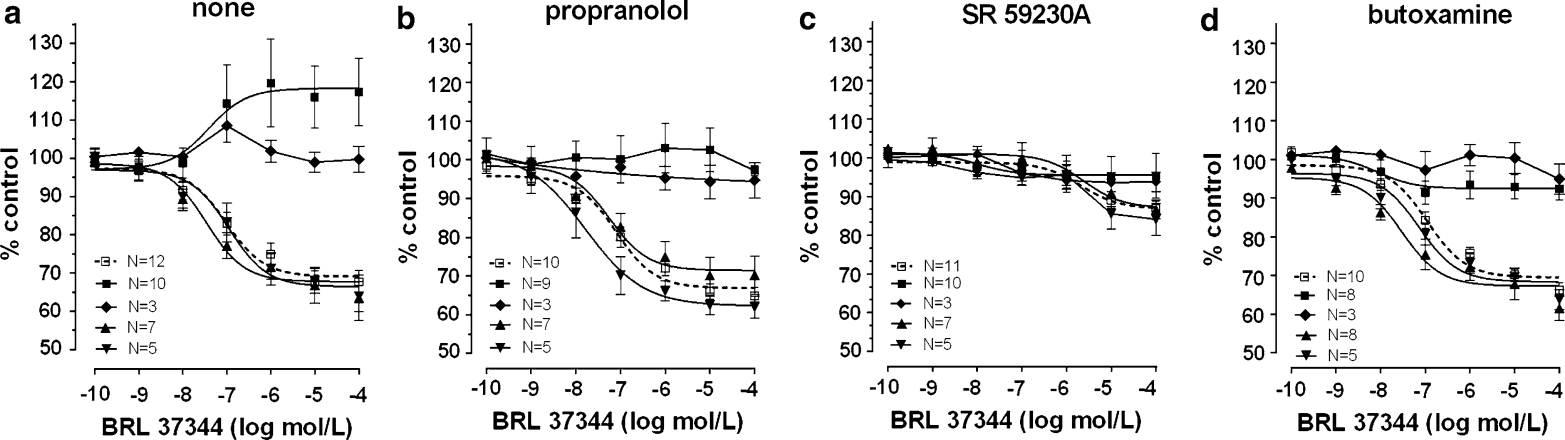
The effect of cumulative administration of BRL 37344 on spontaneous uterine contractility of the human non-pregnant myometrium alone (**a**) and when pre-incubated with propranolol (**b**), SR 59230A (**c**), or butoxamine (**d**), as measured by the area under the curve. (*open square*)—reference group, (*filled square*)—ovarian cancer, (*filled diamond*)—synchronous ovarian–endometrial cancer, (*filled upward triangle*)—endometrial cancer, (*filled downward triangle*)—cervical cancer. Each *point* represents the mean ± SEM of *n* individual myometrial strips from different patients. Spontaneous contractions of the myometrial strips before BRL 37344 administration were considered as a control

**Fig. 5:**
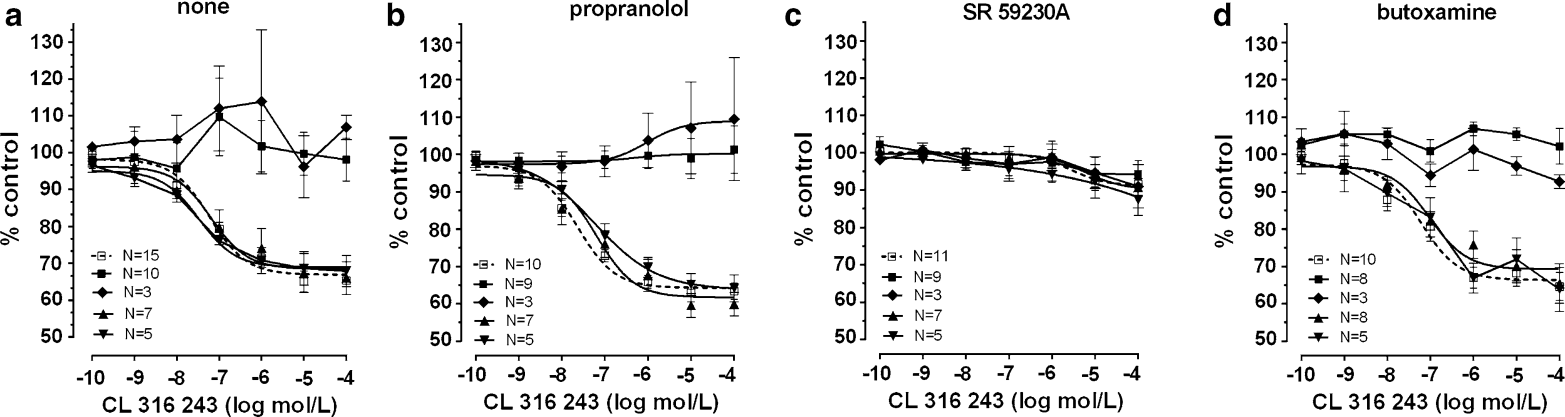
The effect of cumulative administration of Cl 316,243 on spontaneous uterine contractility of the human non-pregnant myometrium alone (**a**) and when pre-incubated with propranolol (**b**), SR 59230A (**c**), or butoxamine (**d**), as measured by the area under the curve. (*open square*)—reference group, (*filled square*)—ovarian cancer, (*filled diamond*)—synchronous ovarian–endometrial cancer, (*filled upward triangle*)—endometrial cancer, (*filled downward triangle*)—cervical cancer. Each *point* represents the mean ± SEM of *n* individual myometrial strips from different patients. Spontaneous contractions of the myometrial strips before Cl 316,243 administration were considered as a control

**Fig. 6:**
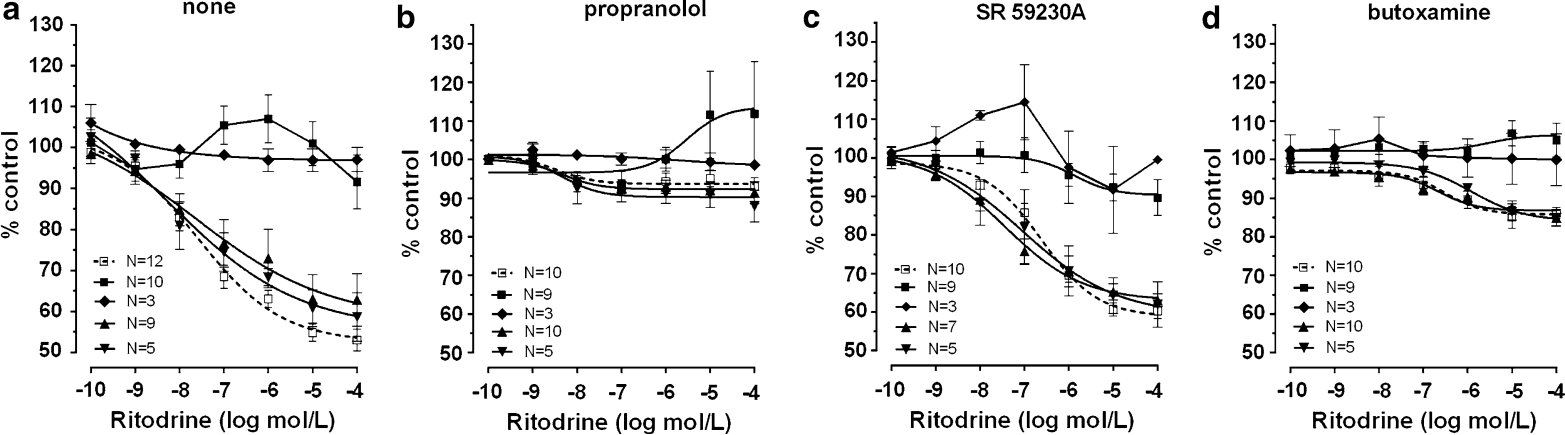
The effect of cumulative administration of ritodrine on spontaneous uterine contractility of the human non-pregnant myometrium alone (**a**) and when pre-incubated with propranolol (**b**), SR 59230A (**c**), or butoxamine (**d**), as measured by the area under the curve. (*open square*)—reference group, (*filled* square)—ovarian cancer, (*filled diamond*)—synchronous ovarian–endometrial cancer, (*filled upward triangle*)—endometrial cancer, (*filled downward triangle*)—cervical cancer. Each* point* represents the mean ± SEM of *n* individual myometrial strips from different patients. Spontaneous contractions of the myometrial strips before ritodrine administration were considered as a control

In the ovarian cancer or synchronous ovarian–endometrial cancer groups, conversely to the effects observed in the other groups, the presence of propranolol inhibited the changes of contractions induced by BRL 37344, CL 316243, and ritodrine in concentrations higher than 10^−9^ mol/l (*p* < 0.01) (Figs. 4b, 5b, 6b). There was no significant variation in the mean value for log EC_50_ for β_3_-adrenoceptor agonists between these groups, yet a statistically significant (*p* < 0.01) shift to the right of the concentration–response curve for ritodrine in the ovarian and synchronous ovarian–endometrial cancer groups was observed as compared to the reference, endometrial cancer, or cervical cancer groups (Tab.1). The *E*_max_ values for ovarian and synchronous ovarian–endometrial cancers were also significantly higher (*p* < 0.001) than in the other three groups.

#### Sr 59230a

Preincubation of myometrial strips with SR 59230A led to a significant inhibition of the relaxation effects for BRL 37344 or CL 316243 in concentrations higher than 10^−9^ mol/l in the reference and endometrial cancer groups (*p* < 0.05), and higher than 10^−8^ mol/l for cervical cancer (*p* < 0.05) (Figs. 3b, 4c, 5c; Table 1). A statistically significant shift to the right of the concentration–response curve for BRL 37344 (*p* < 0.01) and CL 316243 (*p* < 0.01) was observed in the reference and endometrial cancer groups, but not for cervical cancer (Table 1).

The addition of SR 59230A to the organ bath in the reference and cervical cancer groups caused a shift to the right of the ritodrine-induced uterine contractility relaxation (*p* < 0.0001; and *p* < 0.01, respectively) but did not significantly change the *E*_max_ value. In the endometrial cancer group, there were no changes in log EC_50_ or *E*_max_ values (Fig. 6c; Table 1). Moreover, preincubation with the β_3_-adrenoceptor antagonist revealed a statistically significant shift to the left of concentration–response curve for ritodrine (*p* < 0.05) in the endometrial cancer group as compared with the reference group (Table1).

The addition of SR 59230A inhibited relaxation caused by CL 316243 in the reference and endometrial and cervical cancer groups but did not affect the AUC value in the ovarian and synchronous ovarian–endometrial cancer groups. In contrast, the β_3_-adrenoceptor antagonist significantly (*p* < 0.05) decreased the response of tissues to BRL 37344 in all groups (Figs. 4c, 5c). *E*_max_ values (90.3 ± 3.0%) for the ritodrine-induced uterine contractility changes in the presence of SR 59230A in ovarian cancer were statistically higher than in the reference and endometrial cancer groups (60.5 ± 1.2%, *p* < 0.0001; and 65.9 ± 1.5%, *p* < 0.0001, respectively). Similarly, this parameter for synchronous ovarian–endometrial cancer (94.0 ± 6.3%) was statistically higher than in the reference (*p* < 0.05), and endometrial (*p* < 0.05) and cervical cancer (64.2 ± 1.8%, *p* < 0.51) groups (Table 1).

#### Butoxamine

The AUC as a function of BRL 37344 or CL 316243 concentration indicated that pre-treatment with 10^−6^ mol/l butoxamine did not counteract the β_3_-adrenoceptor agonist-induced relaxation for spontaneous uterine contractility in the reference, and endometrial and cervical cancer groups (Figs. 3c, 4d, 5d; Table 1), a conclusive observation of our previous reports [7, 8].

In these three groups, pre-treatment with butoxamine significantly decreased the relaxation caused by ritodrine (Fig. 6d; Table 1). However, the observed rightward shift of the concentration–response curve was not statistically significant.

In the ovarian and synchronous ovarian–endometrial cancer groups, preincubation with butoxamine did not significantly alter the BRL 37344-, CL 316243-, or ritodrine-induced changes in uterine contractility compared with the effects observed in the absence of β_2_-adrenoceptor antagonist (Figs. 4d, 5d, 6d). The *E*_max_ values for concentration–response curves for all β-adrenoceptor agonists in ovarian cancer were substantially higher than the corresponding values for other groups (*p* < 0.0001), however, there were no significant changes between log EC_50_ in these groups (Table 1). Overall, no relaxation of uterine muscle caused by ritodrine, BRL 37344, or CL 316243 was observed in ovarian and synchronous ovarian–endometrial cancers.

As the final step of the statistical analysis, cases of cervical pre-malignancy demonstrating regular spontaneous contractile activity (*n* = 9) were compared with both the remaining reference cases (*n* = 18) and cervical cancers with such activity (*n* = 5). No statistical differences in uterine contractility parameters were detected.

## Discussion

This study describes previously unreported observations on uterine muscle responses to β-adrenoceptor agonists when malignant neoplastic processes affect the reproductive system. The specimens were collected in a prospective manner. Initially, it was not our intention to include rare synchronous tumors of the genital tract. The most common combination is the existence of synchronous malignancies in the ovary and endometrium [25]. When they appeared during the course of this study, we decided to analyze, rather than discard, these potentially vital data because they could provide additional evidence of altered uterine contractility in response to β-adrenoceptor agonists in ovarian cancer, and, as hoped, they did provide important findings: the results for ovarian and synchronous ovarian–endometrial cancers were mostly uniform and different from other studied groups.

Our data showed that BRL 37344, CL 316243, and ritodrine induced dose-dependent attenuation for uterine contractility in the endometrial and cervical cancer groups, similar to that observed in the reference group. Entirely different effects on uterine contractility were observed after cumulative addition of BRL 37344, CL 316243, or ritodrine in the ovarian and synchronous ovarian–endometrial cancer groups. CL 316243 or ritodrine abolished the relaxation of uterine strips whereas BRL 37344 (solely in the ovarian cancer group) increased the AUC for the concentration–response curve as compared with the reference group. Our previous reports demonstrated that both β_2_- and β_3_-adrenoceptor agonists cause concentration-dependent relaxation during spontaneous contractile activity of the human myometrium in women undergoing hysterectomy for benign conditions [7, 8]. The current results indicate that the response of uterine contractility to β-adrenoceptor agonists is markedly altered when a neoplastic process involves the ovaries.

It is well established that ritodrine selectively stimulates β_2_-adrenoceptors, increases intramyocytic cAMP levels, and decreases intracellular Ca^2+^ concentrations, which leads to the relaxation of uterine smooth muscle [26]. Furthermore, ritodrine has been shown to activate BK_Ca_ channels via G-proteins and the cAMP-dependent phosphorylation cascade in pregnant human myometrium [27]. BK_Ca_ channels, activated by both voltage and increased concentrations of intracellular Ca^2+^, are present in uterine smooth muscle [28] and play a significant role in limiting depolarization, thus reducing uterine contractility. Also, activation of the BK_Ca_ channels may explain the effective uterorelaxant influence of β_3_-adrenoceptor agonists [29]. By integrating numerous inputs from diverse stimuli, the BK_Ca_ channel influences and affects various cellular pathways. The mechanisms by which this signaling is specifically routed are not fully understood [27]. Furthermore, it was reported that in cultured adult ventricular myocytes, the abundance of voltage-gated L-type Ca^2+^ channels is altered by β-adrenergic receptor stimulation and by an elevation of the intracellular Ca^2+^ concentration [30]. Moreover, it has been postulated that in human cardiac myocytes, the β_3_-adrenoceptor can change its signaling pathway from a stimulatory, G_s_-mediated cAMP/PKA mechanism to an inhibitory, G_i/o_-mediated type, depending on the cellular context [31]. These findings beg the question: What causes the different responses of the cAMP/PKA pathway to β-adrenoreceptor agonists in human uterine strips with ovarian cancer?

A tumor mass is not a homogenous entity solely consisting of proliferating cancer cells. In fact, it recruits multiple different types of common, healthy cells to form a tumor-associated matrix for further neoangiogenesis, lymphangiogenesis, and neoneurogenesis [32]. It was reported that cancer cells may express receptors for neurotransmitters but they are also capable of synthesizing numerous neurotransmitters. Some of them are believed to act locally in autocrine and paracrine manners, or systemically circulate to conduct relevant regulation on different cells [33]. The possibility that malignant ovarian cells produce substances changing the expression of β-adrenoceptors or ion channels (such as BK_Ca_ or L-type Ca^2+^) should be taken into consideration. Recently, the role of the β-adrenergic system in cancer development and progression has been examined and described [32, 33]. It has been demonstrated that high levels of stress and, in consequence, elevated levels of adrenaline and noradrenaline were found in ovarian cancer patients, and correlated with tumor grade and stage [17, 34]. Furthermore, Sood and Lutgendorf [1] showed that the adrenergic system efficiently inhibited anoikis, a form of programmed cell death, when human ovarian cancer cells were stimulated by adrenaline or noradrenaline. Moreover, activation of β-adrenoceptors leads to the stimulation of protein kinase A (PKA) via the G protein and cAMP-dependent phosphorylation cascade, which subsequently regulate a wide variety of cellular processes ranging from general metabolism and growth to highly specific processes such as differentiation, morphology, motility, secretion, neurotransmission, and gene transcription [2]. Another cAMP effector is the guanine nucleotide exchange protein activated by adenylate cyclase, which influences the induction of genes encoding for cytokines and growth factors complementary to those predominantly mediated by PKA, but with distinct effects on cell morphology and motility [2].

Conversely, membrane ion channels might be responsible for different reactions of uterine contractility to β-adrenoceptor agonists observed in our study. Ion channels are critically important signaling molecules expressed in tissues where they have substantial involvement in determining a diversity of cellular functions. Although ion channels are increasingly being described in cancer cells both in vitro and in vivo, and influence different aspects and stages of carcinogenesis, little is known about the mechanisms controlling their expression. Voltage-gated Na^+^ channels (VGSCs) are functionally expressed in many types of malignant neoplasms of epithelial origin, including those of the breast, skin, colon, cervix, ovary, and prostate, where they promote disease progression and possibly lead to metastasis [35]. Earlier, Fraser et al. (2005) demonstrated significant up-regulation of VGSCs in human breast cancer and proposed neonatal Na_V_1.5 as a novel marker for a metastatic phenotype and therapeutic target. Furthermore, overexpression of Na_V_1.5 was found to play an important role in the progression of ovarian cancer to the metastatic stage [36].

One predominant feature of β-adrenoceptor agonists is their ability to activate BK_Ca_ channels, thus causing hyperpolarization of cellular membranes [27, 29]. It is likely that the altered response of the myometrium to these agonists in cases of ovarian cancer, as evidenced in the present study, may be a consequence of the changed or modulated function of BK_Ca_ channels. A systematic analysis of the entire pool of ion channels and transporters involved is essential to completely understand the role of ionic activity in ovarian cancer and exploit this knowledge clinically.

Preincubation of the uterine strips with propranolol in the reference, and endometrial and cervical cancer groups counteracted the relaxation effects induced by ritodrine but not BRL37344 or CL 316243. In the ovarian and synchronous ovarian–endometrial cancer groups, β_3_-adrenoceptor agonists did not significantly alter uterine contractility while ritodrine did. The presence of SR 59230A led to a significant inhibition of the relaxation effects of all used β-adrenoceptor agonists. Preincubation of uterine muscle strips from women with ovarian or synchronous ovarian–endometrial cancer with β_3_-adrenoceptor antagonist significantly affected the AUC for the concentration–response curve with BRL 37344 or ritodrine, but not CL 316243. The blockage of β_2_-adrenoceptors with butoxamine considerably inhibited the relaxation effects of ritodrine and did not counteract those of β_3_-adrenoceptor agonists in the reference, and endometrial and cervical cancer groups. Butoxamine significantly altered all β-adrenoceptor agonist-induced changes in uterine contractility in both ovarian cancer and synchronous ovarian–endometrial cancer. All in all, a strong influence of the β-adrenoceptor system was observed in the reference, endometrial cancer and cervical cancer groups, whereas dysregulation was present in cases of ovarian and synchronous ovarian–endometrial cancer.

Although based on a large number of observations and under strict histopathological control, the current study has a rather functional character. Electrophysiological testing supplemented by immunocytochemistry and immunohistochemistry may help answer the question of how exactly ovarian cancer dysregulates uterine contractility, regardless of the influence of metastatic lesions [21].

Concisely, the present set of studies demonstrated that ovarian cancer, alone or in combination with endometrial cancer, substantially alters uterine contractility in response to β-adrenoceptor agonists. This new and interesting observation underlines the importance of adrenergic pathways in gynecological, especially ovarian, malignancies and requires further elucidation to better understand how stress hormones affect cancer initiation, growth, and metastatic processes.

## Electronic supplementary material

Below is the link to the electronic supplementary material.

Supplementary material 1 (DOCX 102 kb)

Supplementary material 2 (PDF 240 kb)
