# Supplementary material for: Altered uterine contractility in response to β-adrenoceptor agonists in ovarian cancer
Source: J Physiol Sci. 2016 Nov 12;67(6):711–22. doi: 10.1007/s12576-016-0500-1 (PMC5639028; doi:10.1007/s12576-016-0500-1)
Supplement: Supplementary file 1 — Supplementary material 1 (DOCX 102 kb) [file 12576_2016_500_MOESM1_ESM.docx]

Table 2. Characteristics of β-adrenergic receptors (beta adrenergic receptors, or ADRBs). A - adrenaline, NA - noradrenaline, ISO - isoproterenol.

| Receptor Subtype | β_1_-adrenergic receptor  *also known as*  beta 1 adrenergic receptor,  ADRB1 | β_2_-adrenergic receptor  *also known as*  beta 2 adrenergic receptor,  ADRB2 | β_3_-adrenergic receptor  *also known as*  beta 3 adrenergic receptor,  ADRB3 |
| --- | --- | --- | --- |
| **Gene Coding** | Protein coding for 477 amino acids located on chromosome 10, 10q24-q26 [1] | Protein coding for 413 amino acids located on chromosome 5, 5q31-q32 [2] | Protein coding for 408 amino acids located on chromosome 8, 8p12 [3] |
| **Expression/**  **Localization** | Predominantly, in cardiac tissue [4, 5]. | **Uterus** [6, 7]**.**  Gastrointestinal tract (decreases motility) [8].  Detrusor muscle in the urinary bladder [9].  Bronchi [10].  Blood vessels [10].  Striated muscle [11].  Pancreas [11].  Cerebellum [10]. | Brown adipose tissue [12–14].  Brain (hippocampus, hypothalamus, amygdala, cerebral cortex) [12, 13].  Gut [12, 13]:   - Vascular and non-vascular smooth muscle; - Enteric nervous system; - Neuroendocrine cells.   Urinary bladder [12, 13].  Blood vessels (endothelium) [12, 13].  Myocardium [12, 13].  **Myometrium** [15]**.**  Liver and portal circulation [12, 13]. |
| **Mechanism** | β_1_-adrenergic receptor is linked to Gs proteins which in turn are linked to adenylate cyclase. Agonist binding thus causes a rise in the intracellular concentration of the second messenger cAMP [16–18]. | β_2_-adrenergic receptor is linked to Gs and Gi proteins which in turn are linked to adenylate cyclase. Agonist binding thus causes a rise in the intracellular concentration of the second messenger cAMP. Then protein kinase A, or PKA, is activated and it counterbalances actions of phosphatase PP2A [16–18]. | β_3_-adrenergic receptor is linked to Gs proteins which in turn are linked to adenylate cyclase. Agonist binding thus causes a rise in the intracellular concentration of the second messenger cAMP [8, 18]. |
| **Principal functions of such receptors** | Increase cardiac output [19, 20].  Increase heart rate in sinoatrial node (SA node) [19].  Increase atrial cardiac muscle contractility [19].  Increase contractility and automaticity of ventricular cardiac muscle [21].  Increase conduction and automaticity of atrioventricular node (AV node) [19].  Stimulate production of viscous, amylase-filled secretions from the salivary glands [22].  Activate renin release from juxtaglomerular cells [23].  Trigger lipolysis in adipose tissue [24]. | **Relax smooth muscle** [8, 25, 26]**.**  Increase mass and contraction speed of striated muscle [27].  Activate glycogenolysis and gluconeogenesis in the liver [28]  Stimulate insulin secretion from the pancreas [28, 29].  Thicken secretions from the salivary glands.  In the circulatory system [21, 30–32]:   - decrease cardiac output; - decrease heart rate in SA node; - decrease atrial cardiac muscle contractility; - decrease contractility and automaticity of ventricular cardiac muscle; - dilate the hepatic artery; - dilate arterioles to skeletal muscle.   Inhibit histamine release from mast cells [33].  Increase protein content of secretions from the lacrimal glands [34].  Increase renin secretion from the kidneys [35].  Stimulate bronchiole dilation [25].  Are involved in brain-immune system communication [21]. | Regulate lipolysis and [thermogenesis](https://en.wikipedia.org/wiki/Thermogenesis) [14, 37].  Exert antistress effects in animal studies [37].  **Relax smooth muscle** [12, 15, 37]. |
| **Natural Agonists** | A, NA [38] | A [38] | A, NA [38] |
| **Synthetic Agonists** | ISO [39],  Denopamine [40],  Dobutamine [40],  Xamoterol [40],  T-0509 [39]. | **Ritodrine** [41],  ISO [39],  Salbutamol (Albuterol in USA) [42],  Terbutaline [42],  Bitolterol mesylate [43],  Levosalbutamol (Levalbuteral in USA) [44],  Orciprenaline sulfate [45]. | ISO [39],  Amibegron (SR 58611A) [46],  **CL 316243** [47],  **BRL 37344** [47, 48]**,**  L 742791 [49],  L 796568 [50],  LY 368842 [51],  Mirabegron (YM 178) [52],  Ro40-2148 [53],  Solabegron (GW 427353) [54]. |
| **Non-Specific Beta-Blocker Examples** | **Propranolol** [42],  Timolol [55],  Nadolol [47]. | **Propranolol** [42],  Timolol [55],  Nadolol [47]. | **Propranolol** [42],  Timolol [55],  Nadolol [47]. |
| Specific Beta-Blocker Examples | Acebutolol [56],  Atenolol [42],  Betaxolol [57],  Carvedilol [48],  Esmolol [58],  Metoprolol [47],  Nebivolol [59],  Vortioxetine [60]. | Butoxamine [61],  ICI-118551 [62]. | SR 59230A [48],  L-748328 [48],  L-748337 [48]. |

References

1. Frielle T, Collins S, Daniel KW, Caron MG, Lefkowitz RJ, Kobilka BK (1987) Cloning of the cDNA for the human beta 1-adrenergic receptor. Proc Natl Acad Sci U S A 84:7920–7924.

2. Kobilka BK, Dixon RA, Frielle T, Dohlman HG, Bolanowski MA, Sigal IS, Yang-Feng TL, Francke U, Caron MG, Lefkowitz RJ (1987) cDNA for the human β2-adrenergic receptor: a protein with multiple membrane-spanning domains and encoded by a gene whose chromosomal location is shared with that of the receptor for platelet-derived growth factor. Proc Natl Acad Sci U S A 84:46–50. doi: 10.1073/pnas.84.1.46

3. Granneman JG, Lahners KN, Chaudhry A (1991) Molecular cloning and expression of the rat beta 3-adrenergic receptor. Mol Pharmacol 40:895–899.

4. Hancock AA, DeLean AL, Lefkowitz RJ (1979) Quantitative resolution of beta-adrenergic receptor subtypes by selective ligand binding: application of a computerized model fitting technique. Mol Pharmacol 16:1–9.

5. Heinsimer JA, Lefkowitz RJ (1983) The beta-adrenergic receptor in heart failure. Hosp Pract (Off Ed) 18:103–9, 113–7, 121–5.

6. Parida S, Uttam Singh T, Ravi Prakash V, Mishra SK (2013) Molecular and functional characteristics of β3-adrenoceptors in late pregnant mouse uterus: a comparison with β2-adrenoceptors. Eur J Pharmacol 700:74–79. doi: 10.1016/j.ejphar.2012.11.048

7. Verli J, Klukovits A, Kormányos Z, Hajagos-Tóth J, Ducza E, Seres AB, Falkay G, Gáspár R (2013) Uterus-relaxing effect of β2-agonists in combination with phosphodiesterase inhibitors: studies on pregnant rat in vivo and on pregnant human myometrium in vitro. J Obstet Gynaecol Res 39:31–39. doi: 10.1111/j.1447-0756.2012.01929.x

8. Li F, De Godoy M, Rattan S (2004) Role of adenylate and guanylate cyclases in beta1-, beta2-, and beta3-adrenoceptor-mediated relaxation of internal anal sphincter smooth muscle. J Pharmacol Exp Ther 308:1111–1120. doi: 10.1124/jpet.103.060145.is

9. von Heyden B, Riemer RK, Nunes L, Brock GB, Lue TF, Tanagho EA (1995) Response of guinea pig smooth and striated urethral sphincter to cromakalim, prazosin, nifedipine, nitroprusside, and electrical stimulation. Neurourol Urodyn 14:153–168.

10. Minneman KP, Hegstrand LR, Molinoff PB (1979) The pharmacological specificity of beta-1 and beta-2 adrenergic receptors in rat heart and lung in vitro. Mol Pharmacol 16:21–33.

11. André C, Erraji L, Gaston J, Grimber G, Briand P, Guillet JG (1996) Transgenic mice carrying the human beta 2-adrenergic receptor gene with its own promoter overexpress beta 2-adrenergic receptors in liver. Eur J Biochem 241:417–24.

12. Krief S, Lönnqvist F, Raimbault S, Baude B, Van Spronsen A, Arner P, Strosberg AD, Ricquier D, Emorine LJ (1993) Tissue distribution of beta 3-adrenergic receptor mRNA in man. J Clin Invest 91:344–349. doi: 10.1172/JCI116191

13. Berkowitz DE, Nardone NA, Smiley RM, Price DT, Kreutter DK, Fremeau RT, Schwinn DA (1995) Distribution of beta 3-adrenoceptor mRNA in human tissues. Eur J Pharmacol 289:223–228.

14. van der Lans AAJJ, Vosselman MJ, Hanssen MJW, Brans B, van Marken Lichtenbelt WD (2016) Supraclavicular skin temperature and BAT activity in lean healthy adults. J Physiol Sci 66:77–83. doi: 10.1007/s12576-015-0398-z

15. Rouget C, Bardou M, Breuiller-Fouché M, Loustalot C, Qi H, Naline E, Croci T, Cabrol D, Advenier C, Leroy MJ (2005) Beta3-adrenoceptor is the predominant beta-adrenoceptor subtype in human myometrium and its expression is up-regulated in pregnancy. J Clin Endocrinol Metab 90:1644–50. doi: 10.1210/jc.2004-0233

16. Wenzel-Seifert K, Liu HY, Seifert R (2002) Similarities and differences in the coupling of human beta1- and beta2-adrenoceptors to Gs(alpha) splice variants. Biochem Pharmacol 64:9–20.

17. Yatani A, Tajima Y, Green SA (1999) Coupling of beta-adrenergic receptors to cardiac L-type Ca2+ channels: preferential coupling of the beta1 versus beta2 receptor subtype and evidence for PKA-independent activation of the channel. Cell Signal 11:337–342.

18. Treinys R, Bogdelis A, Rimkutė L, Jurevičius J, Skeberdis VA (2015) Differences in the control of basal L-type Ca(2+) current by the cyclic AMP signaling cascade in frog, rat, and human cardiac myocytes. J Physiol Sci 66:327–336. doi: 10.1007/s12576-015-0430-3

19. Rohrer DK, Desai KH, Jasper JR, Stevens ME, Regula DP, Barsh GS, Bernstein D, Kobilka BK (1996) Targeted disruption of the mouse beta1-adrenergic receptor gene: developmental and cardiovascular effects. Proc Natl Acad Sci U S A 93:7375–80.

20. Ranade K, Jorgenson E, Sheu WH-H, Pei D, Hsiung CA, Chiang F, Chen YI, Pratt R, Olshen RA, Curb D, Cox DR, Botstein D, Risch N (2002) A polymorphism in the beta1 adrenergic receptor is associated with resting heart rate. Am J Hum Genet 70:935–942. doi: 10.1086/339621

21. Pönicke K, Heinroth-Hoffmann I, Brodde O-E (2003) Role of beta 1- and beta 2-adrenoceptors in hypertrophic and apoptotic effects of noradrenaline and adrenaline in adult rat ventricular cardiomyocytes. Naunyn Schmiedebergs Arch Pharmacol 367:592–9. doi: 10.1007/s00210-003-0754-z

22. Yeh CK, Chandrasekar B, Lin AL, Dang H, Kamat A, Zhu B, Katz MS (2012) Cellular signals underlying β-adrenergic receptor mediated salivary gland enlargement. Differentiation 83:68–76. doi: 10.1016/j.diff.2011.09.002

23. Brodde OE, Michel MC (1992) Adrenergic receptors and their signal transduction mechanisms in hypertension. J Hypertens Suppl 10:S133-45.

24. Cao DX, Wu GH, Yang ZA et al (2010) Role of beta1-adrenoceptor in increased lipolysis in cancer cachexia. Cancer Sci 101:1639–1645. doi: 10.1111/j.1349-7006.2010.01582.x

25. Callaerts-Vegh Z, Evans KLJ, Dudekula N, Cuba D, Knoll BJ, Callaerts PFK, Giles H, Shardonofsky FR, Bond RA (2004) Effects of acute and chronic administration of beta-adrenoceptor ligands on airway function in a murine model of asthma. Proc Natl Acad Sci U S A 101:4948–4953. doi: 10.1073/pnas.0400452101

26. Liu YL, Nwosu UC, Rice PJ (1998) Relaxation of isolated human myometrial muscle by beta2-adrenergic receptors but not beta1-adrenergic receptors. Am J Obstet Gynecol 179:895–8.

27. Cairns SP, Borrani F (2015) β-Adrenergic modulation of skeletal muscle contraction: key role of excitation-contraction coupling. J Physiol 593:4713–4727. doi: 10.1113/JP270909

28. Ciccarelli M, Santulli G, Pascale V, Trimarco B, Iaccarino G (2013) Adrenergic receptors and metabolism: Role in development of cardiovascular disease. Front Physiol 4 OCT:265. doi: 10.3389/fphys.2013.00265

29. Ng KW (2011) Regulation of glucose metabolism and the skeleton. Clin Endocrinol (Oxf) 75:147–155. doi: 10.1111/j.1365-2265.2011.04133.x

30. Heubach JF, Trebess I, Wettwer E, Himmel HM, Michel MC, Kaumann AJ, Koch WJ, Harding SE, Ravens U (1999) L-type calcium current and contractility in ventricular myocytes from mice overexpressing the cardiac beta 2-adrenoceptor. Cardiovasc Res 42:173–182.

31. Chruscinski AJ, Rohrer DK, Schauble E, Desai KH, Bernstein D, Kobilka BK (1999) Targeted Disruption of the beta 2 Adrenergic Receptor Gene. J Biol Chem 274:16694–16700. doi: 10.1074/jbc.274.24.16694

32. Rohrer DK, Chruscinski A, Schauble EH, Bernstein D, Kobilka BK (1999) Cardiovascular and metabolic alterations in mice lacking both beta1- and beta2-adrenergic receptors. J Biol Chem 274:16701–16708. doi: 10.1016/S1050-1738(02)00176-7

33. Jones H, Hargrove L, Kennedy L, Meng F, Graf-Eaton A, Owens J, Alpini G, Johnson C, Bernuzzi F, Demieville J, DeMorrow S, Invernizzi P, Francis H (2016) Inhibition of mast cell-secreted histamine decreases biliary proliferation and fibrosis in primary sclerosing cholangitis Mdr2−/− mice. Hepatology 64:1202–1216. doi: 10.1002/hep.28704

34. Allansmith M, Gudmondsson O., Hann L., Keys C, Bloch KJ, Taubman MA, Sullivan DA (1987) The immune response of the lacrimal gland to antigenic exposure. Curr eye … 6:921–927. doi: 10.3109/02713688709034860

35. Kobori H, Nangaku M, Navar LG, Nishiyama A (2007) The intrarenal renin-angiotensin system: from physiology to the pathobiology of hypertension and kidney disease. Pharmacol Rev 59:251–287. doi: 10.1124/pr.59.3.3

36. Andersson D, Wahrenberg H, Löfgren P (2009) Beta3-adrenoceptor function and long-term changes in body weight. Int J Obes (Lond) 33:662–668. doi: 10.1038/ijo.2009.54

37. Sawa M, Harada H (2006) Recent developments in the design of orally bioavailable beta3-adrenergic receptor agonists. Curr Med Chem 13:25–37.

38. Frielle T, Daniel KW, Caron MG, Lefkowitz RJ (1988) Structural basis of beta-adrenergic receptor subtype specificity studied with chimeric beta 1/beta 2-adrenergic receptors. Proc Natl Acad Sci U S A 85:9494–9498.

39. Sato Y, Kurose H, Isogaya M, Nagao T (1996) Molecular characterization of pharmacological properties of T-0509 for beta-adrenoceptors. Eur J Pharmacol 315:363–7.

40. Isogaya M, Sugimoto Y, Tanimura R, Tanaka R, Kikkawa H, Nagao T, Kurose H (1999) Binding pockets of the beta(1)- and beta(2)-adrenergic receptors for subtype-selective agonists. Mol Pharmacol 56:875–885.

41. Park JY, Lee NR, Lee KE, Park S, Kim YJ, Gwak HS (2014) Effects of β2-adrenergic receptor gene polymorphisms on ritodrine therapy in pregnant women with preterm labor: prospective follow-up study. Int J Mol Sci 15:12885–12894. doi: 10.3390/ijms150712885

42. Baker JG (2005) The selectivity of β-adrenoceptor antagonists at the human β1, β2 and β3 adrenoceptors. Br J Pharmacol 144:317–322. doi: 10.1038/sj.bjp.0706048

43. Walker SB, Kradjan WA, Bierman CW (1985) Bitolterol mesylate: a beta-adrenergic agent. Chemistry, pharmacokinetics, pharmacodynamics, adverse effects and clinical efficacy in asthma. Pharmacotherapy 5:127–137.

44. Gupta MK, Singh M (2007) Evidence based review on levosalbutamol. Indian J Pediatr 74:161–167. doi: 10.1007/s12098-007-0010-5

45. Soriano-Ursúa MA, Valencia-Hernández I, Arellano-Mendoza MG, Correa-Basurto J, Trujillo-Ferrara JG (2009) Synthesis, pharmacological and in silico evaluation of 1-(4-di-hydroxy-3,5-dioxa-4-borabicyclo[4.4.0]deca-7,9,11-trien-9-yl)-2- (tert-butylamino)ethanol, a compound designed to act as a ?? 2 adrenoceptor agonist. Eur J Med Chem 44:2840–2846. doi: 10.1016/j.ejmech.2008.12.016

46. Blin N, Camoin L, Maigret B, Strosberg AD (1993) Structural and conformational features determining selective signal transduction in the beta 3-adrenergic receptor. Mol Pharmacol 44:1094–1104.

47. Carpéné C, Galitzky J, Fontana E, Atgié C, Lafontan M, Berlan M (1999) Selective activation of beta3-adrenoceptors by octopamine: comparative studies in mammalian fat cells. Naunyn Schmiedebergs Arch Pharmacol 359:310–321. doi: 10.1007/pl00005357

48. Candelore MR, Deng L, Tota L, Guan X-M, Amend A, Liu Y, Newbold R, Cascieri MA, Weber AE (1999) Potent and Selective Human β3-Adrenergic Receptor Antagonists. J Pharmacol Exp Ther 290:649–655.

49. Weber AE, Ok HO, Alvaro RF, Candelore MR, Cascieri MA, Chiu SHL, Deng L, Forrest MJ, Hom GJ, Hutchins JE, Kao J, MacIntyre DE, Mathvink RJ, McLoughlin D, Miller RR, Newbold RC, Olah T V, Parmee ER, Perkins L, Stearns RA, Strader CD, Szumiloski J, Tang YS, Tola L, Vicario PP, Wyvratt MJ, Fisher MH (1998) 3-Pyridyloxypropanolamine agonists of the beta 3 adrenergic receptor with improved pharmacokinetic properties. Bioorganic Med Chem Lett 8:2111–2116. doi: 10.1016/S0960-894X(98)00381-3

50. Dow RL, Paight ES, Schneider SR, Hadcock JR, Hargrove DM, Martin KA, Maurer TS, Nardone NA, Tess DA, DaSilva-Jardine P (2004) Potent and selective, sulfamide-based human β3-adrenergic receptor agonists. Bioorganic Med Chem Lett 14:3235–3240. doi: 10.1016/j.bmcl.2004.03.089

51. Abraham TL, Lindsay TJ, Chay SH, Czeskis BA, He MM (2005) Metabolism and disposition of a beta 3-adrenergic receptor agonist LY 368842 in male Fisher 344 rats. Xenobiotica 35:647–660. doi: 10.1080/00498250500187992

52. Takasu T, Ukai M, Sato S, Matsui T, Nagase I, Maruyama T, Sasamata M, Miyata K, Uchida H, Yamaguchi O (2007) Effect of (R)-2-(2-aminothiazol-4-yl)-4’-{2-[(2-hydroxy-2-phenylethyl)amino]ethyl} acetanilide (YM178), a novel selective beta3-adrenoceptor agonist, on bladder function. J Pharmacol Exp Ther 321:642–647. doi: 10.1124/jpet.106.115840

53. Carlisle HJ, Stock MJ (1991) Effect of conventional (mixed beta 1/beta 2) and novel (beta 3) adrenergic agonists on thermoregulatory behavior. Pharmacol Biochem Behav 40:249–54.

54. Hertzberg R, Monreal Santiago G, Moberg C (2015) Synthesis of the β3-adrenergic receptor agonist solabegron and analogous N-(2-ethylamino)-β-amino alcohols from O-acylated cyanohydrins - expanding the scope of minor enantiomer recycling. J Org Chem 80:2937–2941. doi: 10.1021/acs.joc.5b00322

55. Taira CA, Monczor F, Höcht C (2010) Measurement of inverse agonism in β-adrenoceptors. Methods Enzymol 485:37–60. doi: 10.1016/B978-0-12-381296-4.00003-8

56. Baker JG, Hall IP, Hill SJ (2003) Agonist actions of “beta-blockers” provide evidence for two agonist activation sites or conformations of the human beta1-adrenoceptor. Mol Pharmacol 63:1312–1321. doi: 10.1124/mol.63.6.1312

57. Louis SN, Nero TL, Iakovidis D, Jackman GP, Louis WJ (1999) LK 204-545, a highly selective beta1-adrenoceptor antagonist at human beta-adrenoceptors. Eur J Pharmacol 367:431–435.

58. Nasrollahi-Shirazi S, Sucic S, Yang Q, Freissmuth M, Nanoff C (2016) Comparison of the β-Adrenergic Receptor Antagonists Landiolol and Esmolol: Receptor Selectivity, Partial Agonism, and Pharmacochaperoning Actions. J Pharmacol Exp Ther 359:73–81. doi: 10.1124/jpet.116.232884

59. Pauwels PJ, Gommeren W, Van Lommen G, Janssen PA, Leysen JE (1988) The receptor binding profile of the new antihypertensive agent nebivolol and its stereoisomers compared with various beta-adrenergic blockers. Mol Pharmacol 34:843–851.

60. Meeker AS, Herink MC, Haxby DG, Hartung DM (2015) The safety and efficacy of vortioxetine for acute treatment of major depressive disorder: a systematic review and meta-analysis. Syst Rev 4:1–16. doi: 10.1186/s13643-015-0001-y

61. Arai M, Sato T, Takeuchi S, Goto S, Togari A (2013) Dose effects of butoxamine, a selective β2-adrenoceptor antagonist, on bone metabolism in spontaneously hypertensive rat. Eur J Pharmacol 701:7–13. doi: 10.1016/j.ejphar.2012.12.016

62. Gong H, Sun H, Koch WJ, Rau T, Eschenhagen T, Ravens U, Heubach JF, Adamson DL, Harding SE (2002) Specific beta(2)AR blocker ICI 118,551 actively decreases contraction through a G(i)-coupled form of the beta(2)AR in myocytes from failing human heart. Circulation 105:2497–2503. doi: 10.1161/01.CIR.0000017187.61348.95
