# Supplementary material for: Altered uterine contractility in response to β-adrenoceptor agonists in ovarian cancer
Source: J Physiol Sci. 2016 Nov 12;67(6):711–22. doi: 10.1007/s12576-016-0500-1 (PMC5639028; doi:10.1007/s12576-016-0500-1)
Supplement: Supplementary file 2 — Supplementary material 2 (PDF 240 kb) [file 12576_2016_500_MOESM2_ESM.pdf]

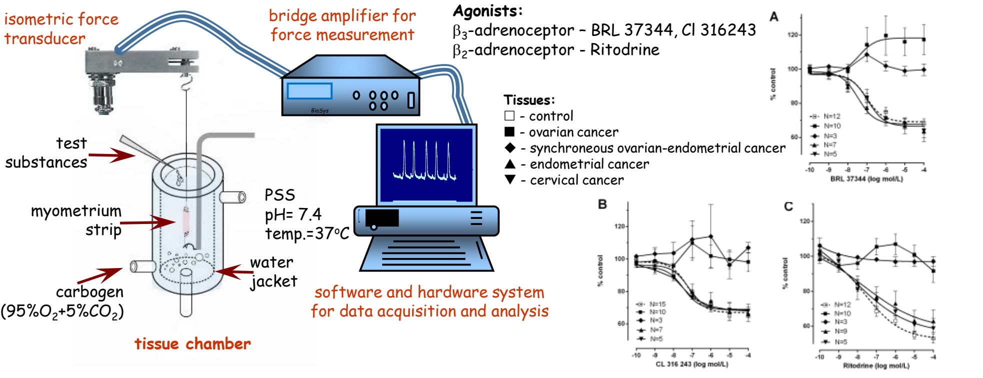

isometric force transducer
bridge amplifier for force measurement
BioSys
test substances
myometrium strip
carbogen
(95%O2+5%CO2)
water jacket
PSS
pH= 7.4
temp.=37°C
tissue chamber
software and hardware system for data acquisition and analysis
Agonists:
β3-adrenoceptor – BRL 37344, Cl 316243
β2-adrenoceptor - Ritodrine
Tissues:
□ - control
■ - ovarian cancer
◆ - synchroneous ovarian-endometrial cancer
▲ - endometrial cancer
▼ - cervical cancer
A
% control
N=12
N=10
N=3
N=7
N=5
BRL 37344 (log mol/L)
B
% control
N=15
N=10
N=3
N=7
N=5
CL 316 243 (log mol/L)
C
% control
N=12
N=10
N=3
N=9
N=5
Ritodrine (log mol/L)
